# Phylogeographical Analyses and Antibiotic Resistance Genes of *Acinetobacter johnsonii* Highlight Its Clinical Relevance

**DOI:** 10.1128/mSphere.00581-20

**Published:** 2020-07-01

**Authors:** Santiago Castillo-Ramírez, Valeria Mateo-Estrada, Gerardo Gonzalez-Rocha, Andrés Opazo-Capurro

**Affiliations:** a Programa de Genomica Evolutiva, Centro de Ciencias Genomicas, Universidad Nacional Autonoma de Mexico, Cuernavaca, México; b Laboratorio de Investigación en Agentes Antibacterianos, Facultad de Ciencias Biológicas, Universidad de Concepción, Concepción, Chile; c Millennium Nucleus for Collaborative Research on Bacterial Resistance, MICROB-R, Concepción, Chile; Antimicrobial Development Specialists, LLC

**Keywords:** *Acinetobacter johnsonii*, population structure, antibiotic resistance, emerging pathogens, horizontal gene transfer, population genomics

## Abstract

Acinetobacter johnsonii has been severely understudied and its population structure and the presence of antibiotic resistance genes (ARGs) are very much uncertain. Our phylogeographical analysis shows that intercontinental transmission has occurred frequently and that different lineages are circulating within single countries; notably, clinical and nonclinical strains are not well differentiated from one another. Importantly, in this species recombination is a significant source of single nucleotide polymorphisms. Furthermore, our results show this species could be an important reservoir of ARGs since it has a significant amount of ARGs, and many of them show signals of horizontal gene transfer. Thus, this study clearly points out the clinical importance of *A. johnsonii* and the urgent need to better appreciate its genomic diversity.

## Abstract (precis)

Acinetobacter johnsonii has been severely understudied and its population structure and the presence of antibiotic resistance genes (ARGs) are very much uncertain. Our phylogeographical analysis shows that intercontinental transmission has occurred frequently and that different lineages are circulating within single countries; notably, clinical and nonclinical strains are not well differentiated from one another. Importantly, in this species recombination is a significant source of single nucleotide polymorphisms.

## OBSERVATION

Acinetobacter johnsonii has not been studied as much as A. baumannii, and few studies have been carried out to examine this species. *A. johnsonii* has been found in aquatic sources, human skin, and animals (1, 2). However, it also causes severe human infections (3–6), highlighting its clinical importance. For instance, Turton et al. showed that 1.7% of 690 nonduplicate Acinetobacter isolates associated with bacteremia were *A. johnsonii* (6). Moreover, Cleland et al. identified *A. johnsonii* as a relevant pathogen involved in chronic rhinosinusitis (7), and some studies have described *A. johnsonii* isolates carrying antibiotic resistance genes (ARGs) (3, 8, 9). For example, different carbapenemase genes, such as *bla*_NDM-1_ and *bla*_OXA-58_, have been identified in *A. johnsonii* (3, 9). The first description of *bla*_NDM-1_-positive *A. johnsonii* occurred in two isolates recovered from sewage in China in 2010 (9), and another isolate (also collected from sewage) was found to coproduce the plasmid-encoded carbapenemases NDM-1, OXA-58, and PER-1 (3). Interestingly, NDM-1 has also been found in phages not only in *A. johnsonii* (10) but also in A. baumannii (11). Hence, this species could be a potential reservoir of ARGs against last-line antibiotics, which is particularly worrying since these genes can be transferred to other clinically relevant microorganisms.

Population genomics studies are needed to achieve a better understanding of the phylogeny and the ARGs within *A. johnsonii*. Although two previous studies conducted some comparative genomics analyses of *A. johnsonii* (3, 4), these only considered a very small number of genomes. Thus, our aim was to characterize the phylogeography and ARGs in *A. johnsonii* using all the genomes available to date. The lack of information on *A. johnsonii* is clear; as of 16 March 2020, there were only 31 genomes on the National Center for Biotechnology Information database. We downloaded these genomes (see Table S1 in the supplemental material) and corroborated that they were *A. johnsonii* by conducting an average nucleotide identity (ANI) analysis via OrthoANI (12). All but one genome (UBA3112) belonged to *A. johnsonii* since they shared ANI values higher than the 95% (the cutoff value for species demarcation) when they were compared. Of note, UBA3112 and UBA8888 were not included in downstream analyses because they did not have high-quality genomes according to CheckM (13) (see also the footnote for Table S1).

10.1128/mSphere.00581-20.3 TABLE S1 List of the genomes and their metadata used for this study. BioSample number, source, and country of origin are provided for the isolates. In addition, it is indicated whether the isolate is related to hospital settings or not (HA, fifth column). The last column indicates cluster to which the isolate belonged according to the population structure analysis. Download Table S1, DOCX file, 0.02 MB. Copyright © 2020 Castillo-Ramírez et al. 2020 Castillo-Ramírez et al. This content is distributed under the terms of the Creative Commons Attribution 4.0 International license.

A pangenome analysis through Roary (14) yielded a total of 13,531 groups of homologous genes (GHGs), most of them (89%) within the accessory genome (see Table S2). The strict core genome consisted of 1,538 GHGs and the majority (∼67%) was found in 15% or fewer of the genomes (see Table S2). Moreover, this is an open pangenome (see Fig. S1), since the number of GHGs kept growing as more genomes were considered without tailing off; we therefore did not fully sample the gene repertoire of this species. Then, to evaluate the level of synteny, we conducted a genome alignment considering five genomes (one from each of the clusters identified in the population structure analysis [see below]) using progressiveMauve (15). Figure S2 shows that a significant number of inversions and large-scale changes occurred within these genomes, indicating that this species has undergone a considerable amount of genome rearrangement.

10.1128/mSphere.00581-20.1 FIG S1 Pangenome analysis of *A. johnsonii.* The numbers of core homologous groups (conserved genes) and the total numbers of homologous groups are plotted versus the number of genomes. This is an open pangenome because the total number of homologous groups (dashed line) keeps growing as more genomes are added, without any signal of reaching a plateau. Download FIG S1, PDF file, 0.01 MB. Copyright © 2020 Castillo-Ramírez et al. 2020 Castillo-Ramírez et al. This content is distributed under the terms of the Creative Commons Attribution 4.0 International license.

10.1128/mSphere.00581-20.2 FIG S2 Genome alignment of five *A. johnsonii* genomes, one from each of the clusters defined by the population structure analysis. The alignment was generated using progressiveMauve. Download FIG S2, JPG file, 0.8 MB. Copyright © 2020 Castillo-Ramírez et al. 2020 Castillo-Ramírez et al. This content is distributed under the terms of the Creative Commons Attribution 4.0 International license.

10.1128/mSphere.00581-20.4 TABLE S2 Details about the pangenome analysis. The numbers of genes for the core and accessory genomes are provided. Download Table S2, DOCX file, 0.01 MB. Copyright © 2020 Castillo-Ramírez et al. 2020 Castillo-Ramírez et al. This content is distributed under the terms of the Creative Commons Attribution 4.0 International license.

**FIG 1:**
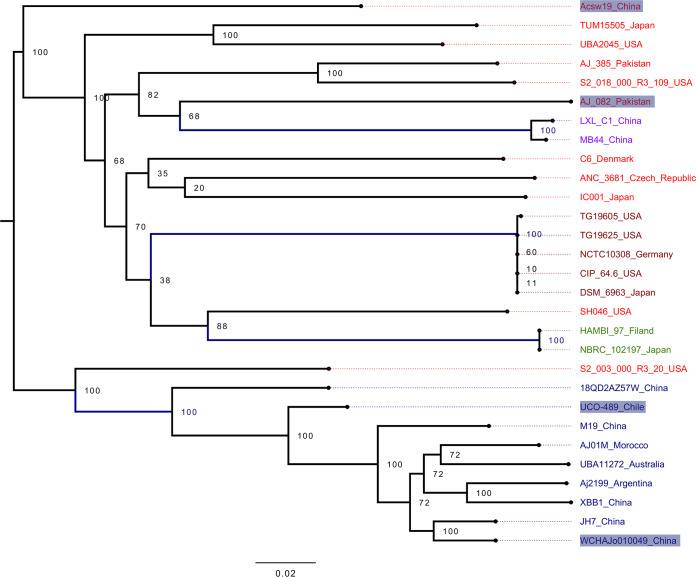
Phylogeny and population structure of *A. johnsonii.* The phylogeny was made on the core genome alignment. Strains are colored according to the clusters found in the population structure analysis and are coded as follows: blue, cluster 1; red, cluster 2; maroon, cluster 3; green, cluster 4; and purple, cluster 5. Gray rectangles denote the isolates having the carbapenemase NDM-1 gene. The numbers by the nodes give the bootstrap values for the nodes, and the scale bar shows the number of substitutions per site.

To establish the population structure and the evolutionary relationships of these isolates, maximum-likelihood phylogeny using PhyML (16) (model GTR+R+I) and population structure analyses via hierBAPS (17) (in Rstudio, with K = 20) were conducted on the core genome alignment, which had 161,087 segregating sites and a nucleotide diversity of 0.0295. We found five genetically differentiated clusters (colored labels in Fig. 1), and four seemed to be real populations, since they were monophyletic groups according to the phylogeny (blue, maroon, green, and purple labels in the figure); in contrast, cluster 2 appeared to be an exclusion group (red labels, Fig. 1). Some of the real clusters had isolates from different continents. For instance, cluster 1 (blue labels) had isolates from South America (Chile and Argentina), Africa (Morocco), Asia (China), and Australia, whereas cluster 3 (maroon labels) contained isolates from Asia (Japan), Europe (Germany), and North America (USA). In addition, different lineages can be circulating in the same country. For instance, isolates from China were found in three of the clusters; this pattern also applies for the Japanese and U.S. strains. Remarkably, we noted that in cluster 2 the clinical isolates (XBB1, Aj2199, and UCO-489) grouped together with environmental isolates such as JH7 (recovered from mine tailings), WCHAJo010049 (collected from sewage), or 18QD2AZ57W (sampled from pig feces).

Thus, these analyses reveal a clear population structure in this species, where some clusters are composed of isolates from distant geographic regions, showing that intercontinental transmission has occurred frequently. Furthermore, different lineages circulate within single countries, implying that several introduction events have happened in the same country. Importantly, there seems to be no clear delimitation between clinical and nonclinical isolates. We used Gubbins (18) to assess the impact of homologous recombination. Clearly, recombination is of paramount importance since the average per-branch recombination/mutation ratio was 4.64, implying that recombination is introducing almost five times more single nucleotide polymorphisms than does mutation.

**FIG 2:**
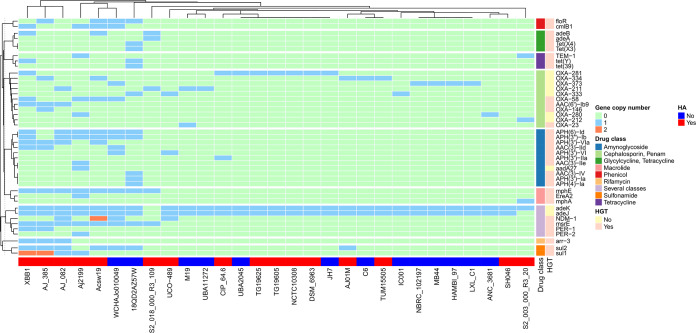
Antibiotic resistance genes in *A. johnsonii*. ARGs in *A. johnsonii* were predicted *in silico*. A heat map shows the frequency of ARGs in the *A. johnsonii* isolates. Antibiotic classes are color-coded. A dendrogram at the top of the figure shows a hierarchical clustering analysis of the strains according to ARG presence. Next to the drug class column, there is a column (HGT key) specifying whether the ARG had identical sequences in other species (salmon) or not (yellow). The row below the heat map indicates whether (red) or not (blue) the isolates are associated with hospitals (HA key).

Finally, we conducted an *in silico* prediction of ARGs by conducting BLAST searches (similarity criteria, ≥80% identity and ≥70% coverage) of the *A. johnsonii* proteomes against the Comprehensive Antibiotic Resistance Database (19). Notably, all the strains, even the environmental ones, had at least two ARGs (see Fig. 2); for instance, isolates C6 and LXL_C1 both had oxacillinases and multidrug efflux resistance-nodulation-division (RND) transporter genes. We found resistant determinants for several drug classes in many isolates (see Fig. 2, drug class). We also looked for mutations conferring resistance to fluoroquinolones via ResFinder (20), but we did not find any. In agreement with previous studies (3, 4), we found some β-lactamase genes (*bla*_NDM-1_, *bla*_PER-1_, *bla*_PER-2_, and *bla*_OXA-58_). In addition to some clinical isolates, two sewage strains (Acsw19 and WCHAJo010049) and a strain collected from pig feces (18QD2AZ57W) had the largest amount of ARGs. In this regard, Tang et al. determined that strain Acsw19 has 12 ARGs in plasmids and in the chromosome (10). Considering the OXA β-lactamases, we found several families: OXA-211-like, OXA-58-like, and OXA-23-like. However, the most abundant—OXA-281, OXA-334 and OXA-373—belong to the OXA-211-like family, which was described rather recently in non-*baumannii* Acinetobacter spp. Remarkably, many ARGs have undergone horizontal gene transfer (HGT) since 81% of them had identical sequences in other bacteria from clinically relevant genera such as *Salmonella*, *Klebsiella*, *Vibrio*, etc. (see Fig. 2 and Table S3). As a case in point, the carbapenemase NDM-1 was present in four isolates (see gray rectangles in Fig. 1) on noncontiguous branches of the tree, implying independent acquisitions of this gene, and identical sequences of this gene were found in many genera other than Acinetobacter (see Table S3). Taken together, these results show that many strains, both clinical and nonclinical, had ARGs with signals of HGT and thus could function as a reservoir of ARGs for other bacteria.

10.1128/mSphere.00581-20.5 TABLE S3 ARGs with identical sequences in other bacteria. All the species that had sequences identical to the ARG in *A. johnsonii* are listed in the organism column. When there were no identical sequences in other species, only *A. johnsonii* is listed in organism column. Download Table S3, XLSX file, 0.01 MB. Copyright © 2020 Castillo-Ramírez et al. 2020 Castillo-Ramírez et al. This content is distributed under the terms of the Creative Commons Attribution 4.0 International license.

In conclusion, we highlight the clinical relevance of this species, since environmental and clinical strains are intermingled with one another, and all the strains show ARGs. Further (genomic and functional) studies of clinical and nonclinical strains are needed to fully understand the clinical nature of this species.
