# Supplementary material for: Phylogeographical Analyses and Antibiotic Resistance Genes of Acinetobacter johnsonii Highlight Its Clinical Relevance
Source: mSphere. 2020 Jul 1;5(4):e00581-20. doi: 10.1128/mSphere.00581-20 (PMC7333577; doi:10.1128/mSphere.00581-20)
Supplement: TABLE S1 [file mSphere.00581-20-st001.docx]

**Table 1 – Isolates and their metadata used in this study.**

All the data for the strains was downloaded from the NCBI. For each strain the Biosample number and the country of origin are provided; also, when possible the source.

| **Strain** | **BioSample** | **Country** | **Source** | **HA^+^** | **Cluster** |
| --- | --- | --- | --- | --- | --- |
| SH046 | SAMN02463729 | USA | NA | YES | 2 |
| TG19605 | SAMN02471272 | USA | duodenum | YES | 3 |
| TG19625 | SAMN02471255 | USA | hospital surface | YES | 3 |
| CIP_64.6 | SAMN01828184 | USA | duodenum | YES | 3 |
| ANC_3681 | SAMN01087830 | Czech Republic | water | NO | 2 |
| DSM_6963 | SAMD00019881 | Japan | duodenum | YES | 3 |
| MB44 | SAMN03571285 | China | plant (*Populus lasiocarpa*) | NO | 5 |
| XBB1 | SAMN03268929 | China | hospital sewage | YES | 1 |
| Aj2199 | SAMN04572947 | Argentina | peritoneal fluid | YES | 1 |
| UBA2045 | SAMN06456972 | USA | metal | NO | 2 |
| UBA3112* | SAMN06452666 | USA | metal/plastic | NO | NA |
| S2_018_000_R3_109 | SAMN07426609 | USA | hospital NICU surfaces and sink | YES | 2 |
| S2_003_000_R3_20 | SAMN07426520 | USA | hospital NICU surfaces and sink | YES | 2 |
| LXL_C1 | SAMN09652504 | China | soil | NO | 5 |
| HAMBI_97 | SAMN09428650 | Finland | environment | NO | 4 |
| UBA8888* | SAMN08017597 | Australia | metagenome | NO | NA |
| UBA11272 | SAMN08020280 | Australia | metagenome | NO | 1 |
| AJ_082 | SAMN10249157 | Pakistan | washroom sink in an ICU | YES | 2 |
| AJ_385 | SAMN10249155 | Pakistan | washroom sink in an ICU | YES | 2 |
| IC001 | SAMN07298690 | Japan | river | NO | 2 |
| WCHAJo010049 | SAMN09499720 | China | sewage | NO | 1 |
| M19 | SAMN11047491 | China | water | NO | 1 |
| NBRC_102197 | SAMD00166014 | Japan | NA | NO | 4 |
| Acsw19 | SAMN12630603 | China | sewage | YES | 2 |
| TUM15505 | SAMD00175240 | Japan | human blood | YES | 2 |
| UCO-489 | SAMN13564663 | Chile | clinical setting | YES | 1 |
| JH7 | SAMN12773519 | China | mine tailings | NO | 1 |
| 18QD2AZ57W | SAMN13531404 | China | pig feces | NO | 1 |
| C6 | SAMEA56643418 | Denmark | water environment | NO | 2 |
| AJ01M | SAMEA104166933 | Morocco | NA | YES | 1 |
| NCTC10308 | SAMEA3936795 | Germany | duodenum | YES | 3 |

*These strains were not included in downstream analyses, as they did not pass the criteria of having high quality genomes. Both showed completeness values as per CheckM below 95%: UBA3112 had a value of 67.4, whereas UBA8888 showed a value of 74.14.

^+^HA: Hospital-associated
