# Supplementary figures and images for: Phylogeographical Analyses and Antibiotic Resistance Genes of Acinetobacter johnsonii Highlight Its Clinical Relevance
Source: mSphere. 2020 Jul 1;5(4):e00581-20. doi: 10.1128/mSphere.00581-20 (PMC7333577; doi:10.1128/mSphere.00581-20)

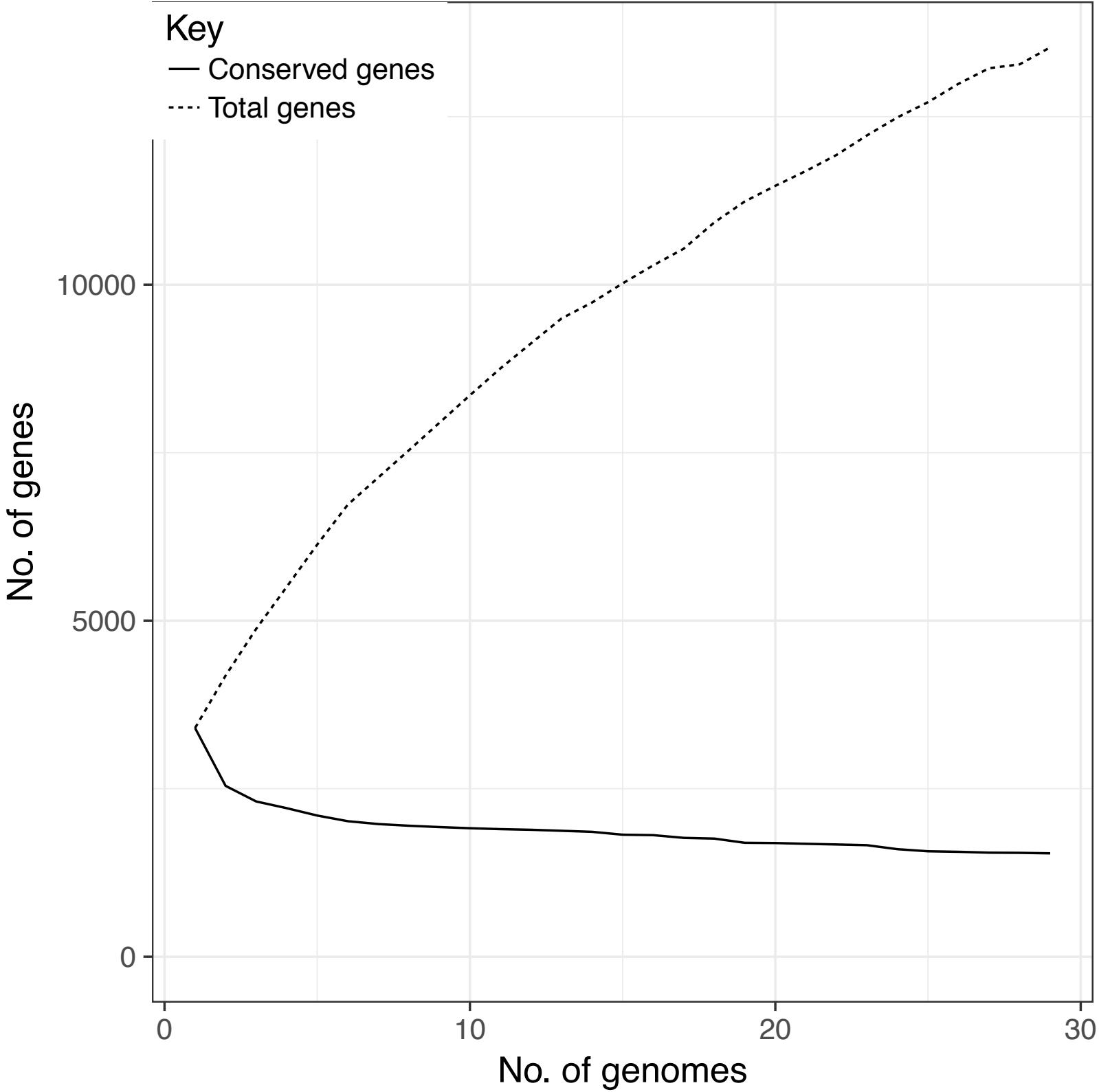

Supplement: FIG S1 [file mSphere.00581-20-sf001.pdf]

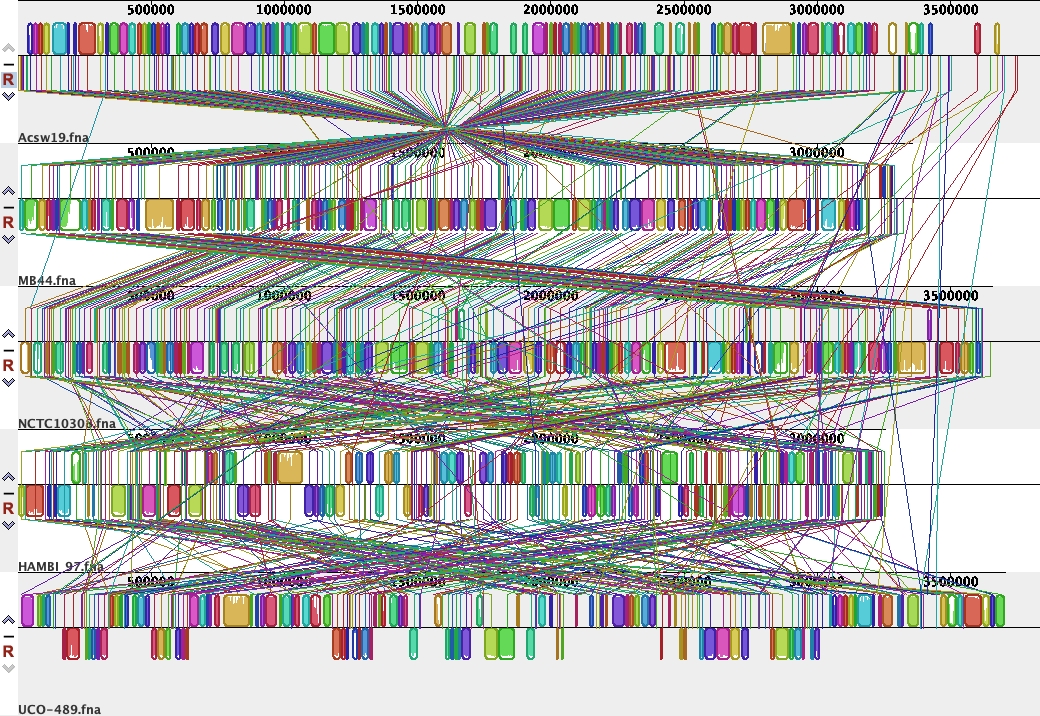

Supplement: FIG S2 [file mSphere.00581-20-sf002.jpg]
