# Supplementary material for: Phylogeographical Analyses and Antibiotic Resistance Genes of Acinetobacter johnsonii Highlight Its Clinical Relevance
Source: mSphere. 2020 Jul 1;5(4):e00581-20. doi: 10.1128/mSphere.00581-20 (PMC7333577; doi:10.1128/mSphere.00581-20)
Supplement: TABLE S2 [file mSphere.00581-20-st002.docx]

**Pangenome analysis**

| **Type of genome** | **Percentage of isolates** | **Number of genes** |
| --- | --- | --- |
| **Core genome** | **100%** | **1538** |
| **Soft core genome** | **95% <= isolates < 100%** | **467** |
| **Shell genome** | **15% <= isolates < 95%** | **2487** |
| **Cloud genome** | **0% <= isolates < 15%** | **9039** |
| **Pan genome** | **0% <= isolates <= 100%** | **13531** |
